# Clinical performance and shape analysis of trifocal intraocular lenses via scanning electron microscopy

**DOI:** 10.1186/s12886-024-03355-3

**Published:** 2024-02-26

**Authors:** Kazuya Yamashita, Koji Hayashi, Seiichiro Hata

**Affiliations:** 1 Department of Ophthalmology, Keiyu Hospital, 3-7-3 Minatomirai, Yokohama Nishi-ku , 220-8521 Yokohama City, Kanagawa Japan; 2 Sky-building Eye Clinic, Yokohama City, Kanagawa Japan

**Keywords:** Trifocal intraocular lens, Contrast sensitivity, Distance-corrected visual acuity, Defocus curve, Scanning electron microscope

## Abstract

**Background:**

To evaluate visual performance after implantation of the TFNT (Acrysof Panoptix, Alcon, Fort Worth, Texas, USA) and CNWT (Clareon Panoptix, Alcon, Fort Worth, Texas, USA) intraocular lens (IOL), and compare the lens shape observed by scanning electron microscope (SEM).

**Methods:**

Eighteen patients (18 eyes) received implantation of the CNWT and Twenty patients (20 eyes) received implantation of the TFNT. Exclusion criteria were previous ocular surgeries, ocular pathologies, or corneal abnormalities. Intervention or Observational Procedure(s): Postoperative examination at 1 months including manifest refraction; evaluation of refractive error, distance-corrected visual acuity (DCVA) at 5 m, 1 m, 70 cm, 50 cm, 40 cm, and 30 cm, slit-lamp examination; defocus curve testing; contrast sensitivity (CS) was performed. The lens shape of the TFNT and the CNWT was examined under SEM.

**Results:**

Mean spherical equivalent was 0.11 ± 0.41 D (CNWT group) and 0.12 ± 0.34 D (TFNT group) 1 month postoperation. DCVA and defocus curve showed no significant difference between the two groups. CS was significantly higher in CNWT group than TFNT group at spatial frequencies of 6 cycles per degree (cpd). Observation of the IOL with a scanning electron microscope (SEM) revealed that CNWT group had improved diffraction structure and edge processing accuracy compared to TFNT group.

**Conclusion:**

There was no significant difference between the two groups in the defocus curve and visual acuity at all distances. CS was better in the CNWT group than in the TFNT group. IOL surface features may affect CS.

## Background

The design of intraocular lens (IOL) is constantly evolving to improve the visual outcome after cataract surgery, and there are different types of IOL. Previously, The TFNT (Acrysof Panoptix, Alcon, Fort Worth, Texas, USA) was able to improve light transmission and distribution among the three focuses, decreasing pupil dependence and improving intermediate vision [1]. CNWT (Clareon Panoptix, Alcon, Fort Worth, Texas, USA) which was released in April 2022, is expected to be an evolved product of TFNT which was released in 2019. CNWT suppresses glistening and Sub-surface nano glistening (SSNG) that can occur after surgery, and maintains the same transparency as immediately after surgery for a long time after surgery [2]. The CNWT provides stable visual acuity as well as superior contrast sensitivity and lower subjective photic phenomena, over the prior IOL [3]. However, the mechanism of better contrast sensitivity was unclear. We compared the visual function results and observed by scanning electron microscope (SEM) after noticing no difference between the TFNT and CNWT groups. To the best of our knowledge, this is the first study to report clinical results for trifocal IOL using comparative results obtained from observation by SEM.

## Methods

### Patients and study design

Our study retrospectively analyzed visual function in individuals slated for the implantation of the specified IOL, employing methodologies akin to those reported in prior research [3]. Complying with the Declaration of Helsinki’s principles, the research protocol received endorsement from the Keiyu Hospital’s ethics committee in Japan, with all participants providing written informed consent. Eligibility for the study required individuals to be at least 20 years of age and potential candidates for cataract surgery, excluding those with any eye conditions potentially impeding visual improvement or with irregular iris structures. Pre-surgical assessments for these candidates encompassed a comprehensive ocular examination: BCVA, anterior segment inspection using biomicroscopy, intraocular pressure assessments, SS-OCT imaging (ANTERION, Heidelberg Engineering GmbH, Heidelberg, Germany), endothelial cell count via specular biomicroscopy (EM-4000, TOMEY CO, LTD, Japan), dilated retinal examination, macula and optic nerve OCT scans (Spectralis OCT, Heidelberg Engineering GmbH, Heidelberg, Germany), and IOL power calculations using the IOL Master 700 (Carl Zeiss Meditec AG, Germany).

### Surgical procedure and postoperative assessment

The surgical approach replicated that of previous protocols [3]. The initial cataract operation was performed on one eye, with the subsequent eye’s surgery scheduled no less than a week later. Follow-up appointments were arranged for the day following each surgery and again one month after the second operation, during which all initial evaluations were repeated. The one-month postoperative visit included a thorough re-examination process that covered monocular defocus curves, mono- and binocular uncorrected visual acuity tests for various distances, subjective refraction, and contrast sensitivity assessments.

Photopic visual acuity measurements were conducted under a room luminance of 85 cd/m2. Participants’ uncorrected visual acuity and distance-corrected visual acuity were gauged using Landolt vision charts. Near visual acuity was measured at distances of 30 and 40 cm, while intermediate visual acuity evaluations took place at 1 m, 70 cm, and 50 cm.

Subjective refraction utilized the Landolt vision charts at a 5 m distance. The defocus curve assessment was then carried out monocularly, with patients viewing the Landolt charts through lenses adjusted from − 1.00 D in 0.50 D increments up to + 3.00 D. Binocular contrast sensitivity was quantified across spatial frequencies of 3, 6, 12, and 18 cpd using the functional acuity contrast test (CSV-1000, Nikon, Co., Ltd, Japan).

The lens shapes of the TFNT and the CNWT were evaluated and photographed under the SEM (JSM-7500 F, JEOL Ltd., Tokyo, Japan). Tissue SEM samples were embedded in epoxy resin at 60 °C for 48 h. Subsequently, the test piece was sectioned using a microtome to prepare a cross-section. Thereafter, the sample was coated with an osmium plasma ion coater, and SEM observation (JSM-7500 F) was performed.

### Statistical analysis

Continuous values were expressed as the mean ± standard deviation. The visual acuity was converted to the logarithm of the minimal angle of resolution (log MAR) values for all calculations. Data are presented as mean ± SD and were compared by the Mann-Whitney U test using the statistical programming language ‘R’ (R version 4.0.2; The Foundation for Statistical Computing, Vienna, Austria). A *p*-value of less than 0.05 was considered statistically significant.

## Results

Age, preoperative subjective refraction values, preoperative corneal astigmatism and the eye axis were collected so that there was no difference between the two groups (Table 1).

**Table 1:** Demographic data on the patients

	CNWT(Clareon PanOptix)	TFNT(AcrySof IQ PanOptix)	*P* value
Patients	18 eyes	20 eyes	-
Age	61 ± 10.1 years	61 ± 10.6 years	*P* = 0.93
Sex	M 3 eyes F 15 eyes	M 11 eyes F 9 eyes	-
preoperative subjective refraction values	-2.90 ± 2.99 D	-2.51 ± 3.77 D	*P* = 0.50
preoperative corneal astigmatism	-1.01 ± 0.67 D	-1.09 ± 0.79 D	*P* = 0.91
eye axis length	24.48 ± 0.91 mm	24.51 ± 0.98 mm	*P* = 0.95

* *P* < 0.05, Wilcoxon Signed rank test

Abbreviation: F: Female, M: Man, L: Left, R: Right

In the CNWT group, the ages ranged from 35 to 72 years, while in the TFNT group, they ranged from 44 to 79 years.

### Visual acuity and refractive status

**Fig. 1:**
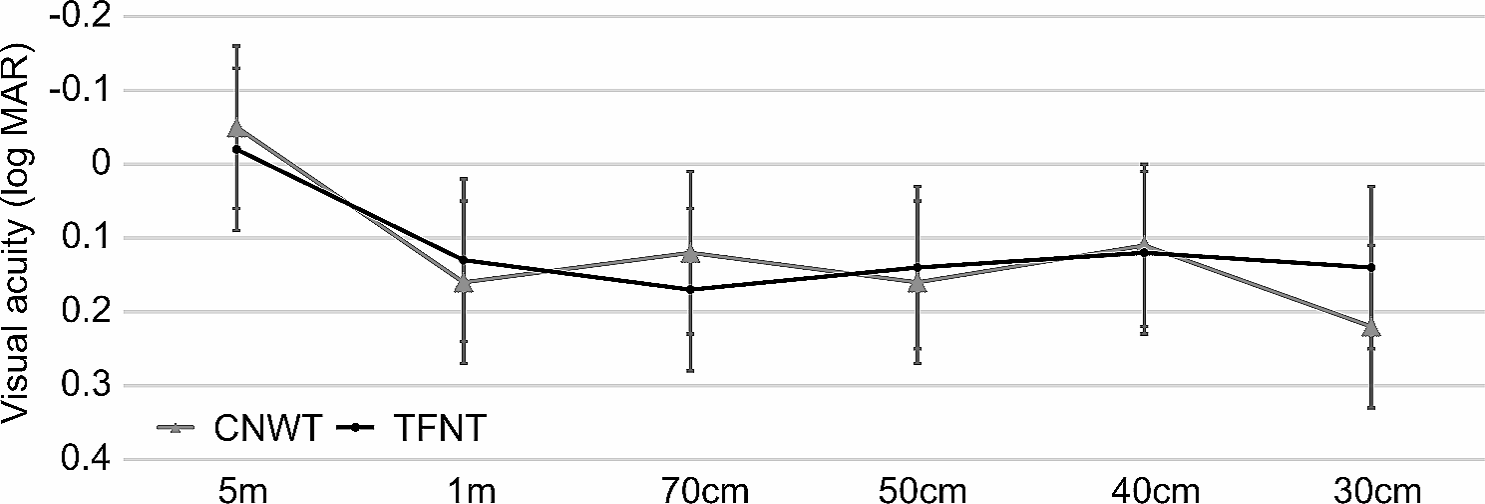
Distance-corrected visual acuity (DCVA) in 5 m, 1 m, 70 cm, 50 cm, 40 cm, and 30 cm. There were no statistically significant differences between the two groups in all distances. LogMAR; logarithm of the minimal angle of resolution

Figure 1 shows uncorrected visual acuity. There were no significant differences between the values in CNWT group and in TFNT group (*p* > 0.05 for all comparisons). One month after surgery, all patients achieved an uncorrected photopic binocular visual acuity of 0.3 LogMAR (Snellen equivalent 20/40) for each distance. For far distances (5 m), all patients achieved an uncorrected photopic binocular visual acuity of 0.0 LogMAR (Snellen equivalent 20/20) or better. There were no significant differences between the CNWT group (-0.05 Log MAR) and the TFNT group (-0.02 Log MAR) (*p* = 0.22). For intermediate distances (1 m, 70 cm, 50 cm), all patients achieved an uncorrected photopic binocular visual acuity of 0.2 LogMAR (Snellen equivalent 20/32) or better. There were no significant differences between the CNWT group (0.16 Log MAR) and the TFNT group (0.13 Log MAR) in the intermediate distance (1 m) (*p* = 0.21), the CNWT group (0.12 Log MAR) and the TFNT group (0.17 Log MAR) in the intermediate distance (70 cm) (*p* = 0.13) and the CNWT group (0.16 Log MAR) and the TFNT group (0.14 Log MAR) in the intermediate distance (50 cm) (*p* = 0.92). For near distances (40 cm and 30 cm), all patients achieved an uncorrected photopic binocular visual acuity of 0.3 LogMAR (Snellen equivalent 20/40) or better. There were no significant differences between the CNWT group (0.11 Log MAR) and the TFNT group (0.12 Log MAR) in the near distance (40 cm) (*p* = 0.81), the CNWT group (0.22 Log MAR) and the TFNT group (0.25 Log MAR) in the near distance (30 cm) (*p* = 0.48). Mean postoperative 1-day spherical equivalent was 0.35 D ± 0.40 (range − 0.05 to + 0.75 D) in CNWT group and 0.43 D ± 0.31 (range 0.12 to + 0.74 D) in TFNT group. Mear postoperative 1-month spherical equivalent was 0.11 D ± 0.38 (range − 0.27 to + 0.49 D) in CNWT group and 0.12 D ± 0.26 (range − 0.14 to + 0.38 D) in TFNT group. There were no significant differences between the values in CNWT group and in TFNT group (*p* > 0.05 for all comparisons).

### Defocus curve

**Fig. 2:**
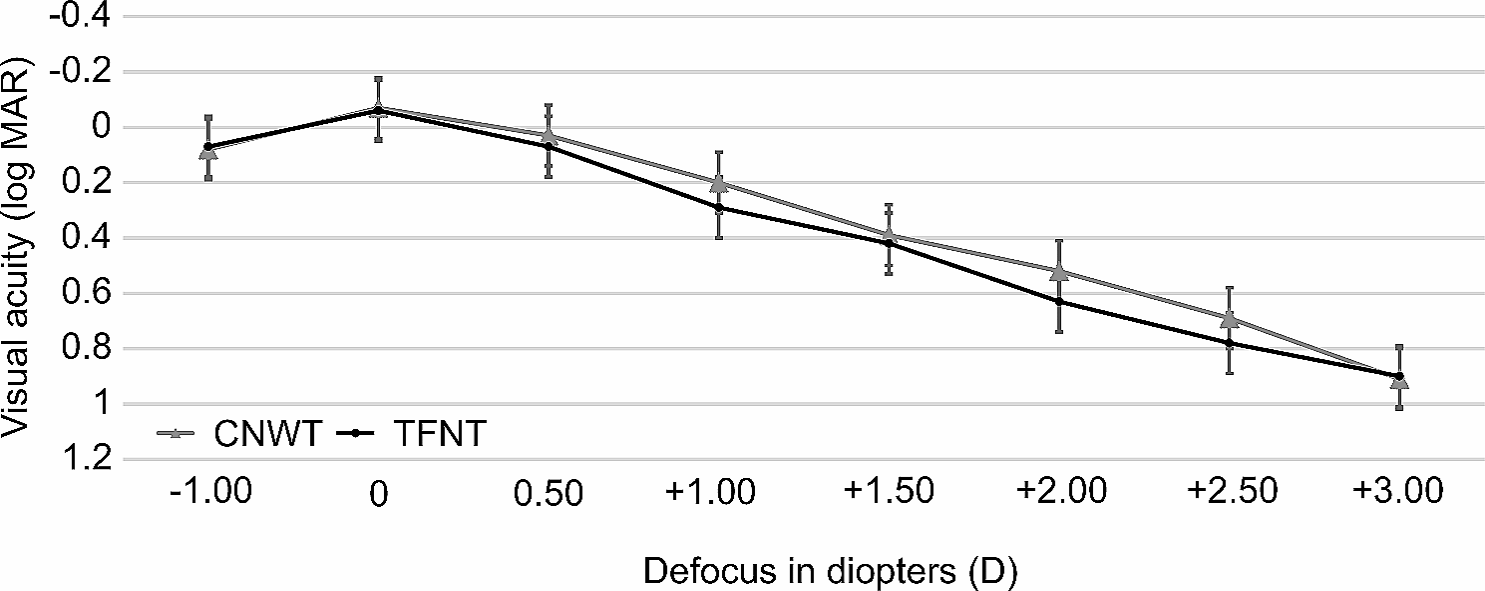
Photopic uncorrected LogMAR monocular defocuses curves of multifocal intraocular lenses with CNWT (Clareon Panoptix) and TFNT (Acrysof Panoptix). There were no statistically significant differences between the two groups in all diopters. D; diopters

Figure 2 depicts the corrected monocular logMAR visual acuity across different vergences. The best visual acuity was achieved at a vergence of 0.00 D, corresponding to far focus, with no significant differences between the CNWT and TFNT groups across various vergences. The visual acuity values and statistical comparisons at different vergences are detailed, indicating a consistent performance between the two groups throughout the defocus curve.

### Contrast sensitivity

**Fig. 3:**
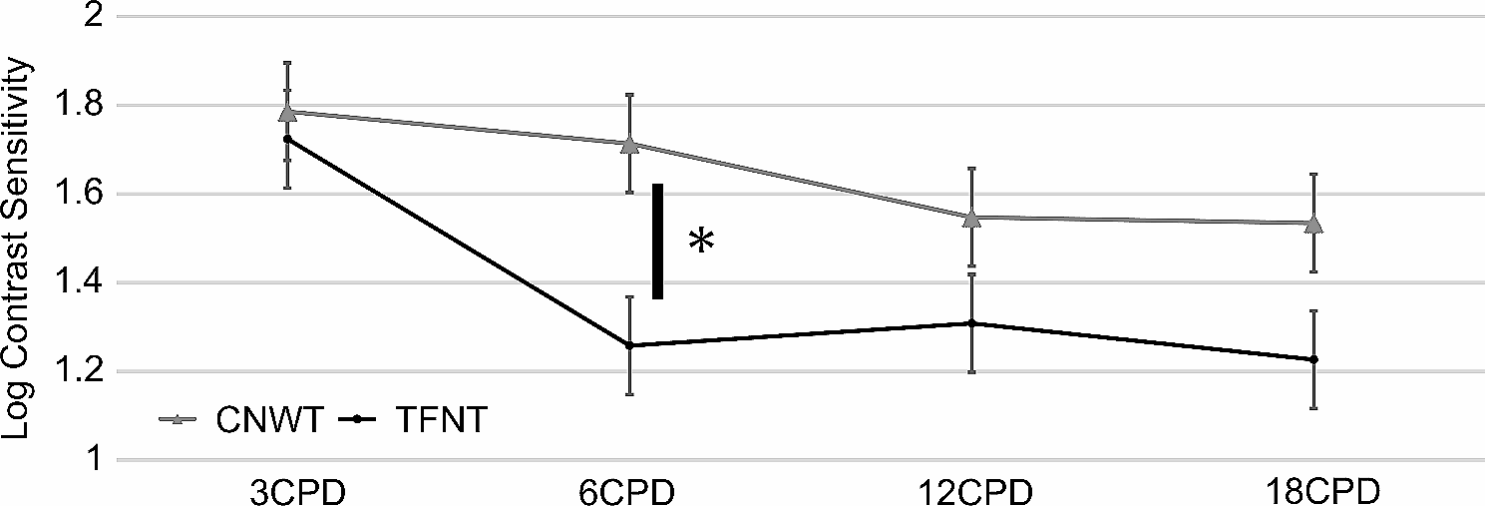
The mean log_10_ contrast sensitivity values under photopic (85 cd/m2) conditions. Contrast sensitivity in CNWT group was higher than in TFNT group in 6 CPD

Figure 3 shows the mean log_10_ contrast sensitivity values under photopic (85 cd/m2) conditions. Contrast sensitivity was similar in 3 cycle per degrees (CPD) (*p* = 0.28), 12 CPD (*p* = 0.17) and 18 CPD (*p* = 0.22). However, Contrast sensitivity in CNWT group was higher than in TFNT group in 6 CPD (*p* = 0.004).

### Scanning electron microscope

**Fig. 4:**
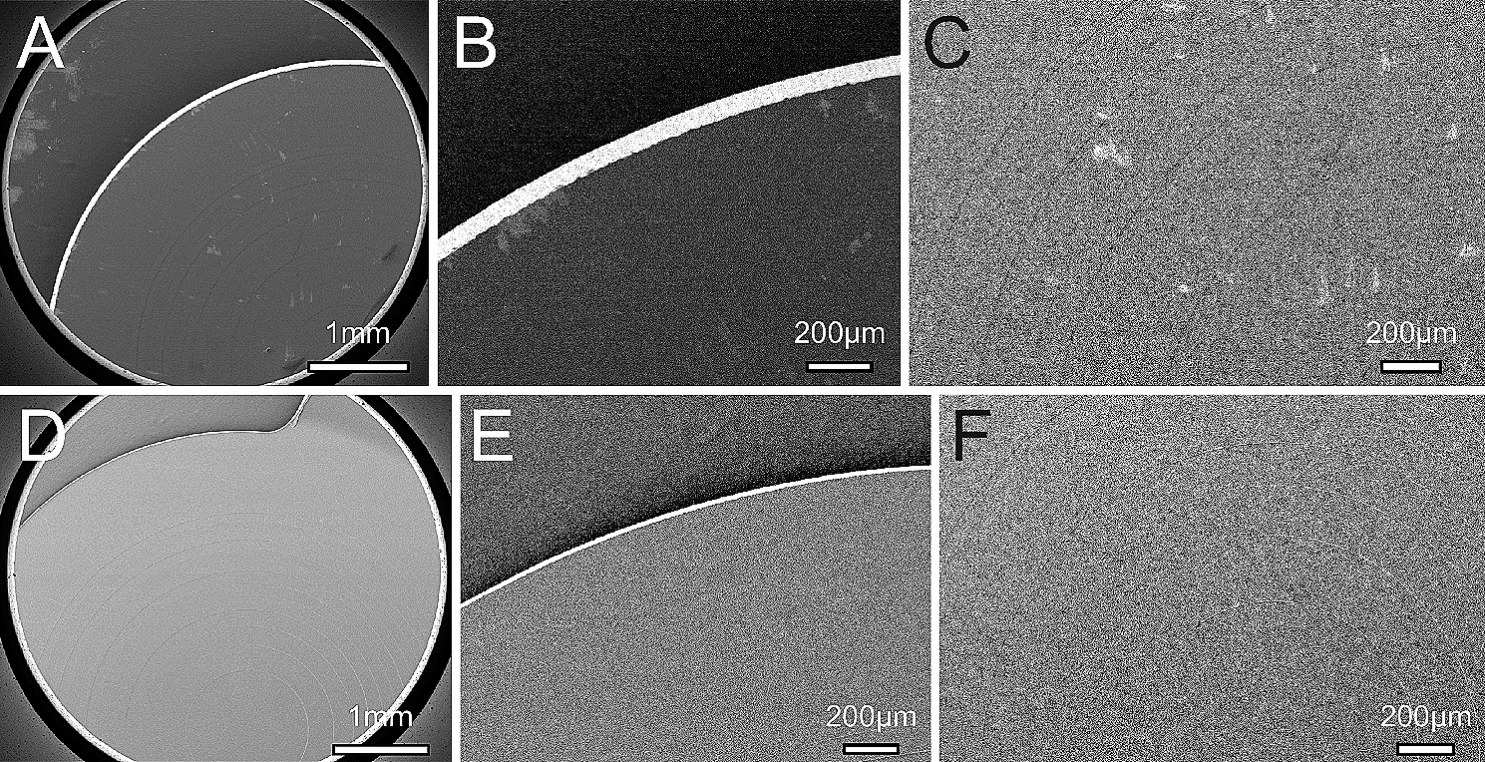
The TFNT (**a**, **b**, **c**) and the CNWT (**d**, **e**, **f**) observed by SEM. The edge of CNWT (**e**) was sharper than the TFNT (**b**), and diffraction fringes of CNWT (**f**) was also clear than the TFNT (**c**)

Figure 4 shows the TFNT (Fig. 4a, b, c) and the CNWT (Fig. 4d, e, f) observed by SEM. The edge of CNWT (Fig. 4e) was sharper than the TFNT (Fig. 4b), and diffraction fringes of CNWT (Fig. 4f) was also clear than the TFNT (Fig. 4c).

## Discussion

SEM is used to view the surface of IOLs and describe a microscopic picture of the IOL surface and edges [4]. IOL surface characteristics including the optic-haptic junction are reported to study by SEM [5]. SEM showed the calcification of hydrophilic IOLs and revealed the exact chemical composition of the deposits [6]. We confirmed that both CNWT and TFNT square-edged IOLs had a curvature radius and the mean optic edge thickness ranged as previously reported [7]. The use of SEM in ophthalmology is quite popular and very helpful in showing a perspective not visible to the naked eye [8].

In our study, we examined the surface characteristics of IOLs using SEM. The TFNT lens demonstrated enhanced clarity and image quality, particularly at intermediate distances [9]. Alcon introduced the Clareon IOL (CNA0T0), utilizing a novel cross-linked acrylic optic biomaterial. This material blends a hydrophilic polymer (2-hydroxyethyl-methacrylate) with a hydrophobic component (phenylethyl acrylate), incorporating a chemically bonded ultraviolet blocker and a blue-light filtering chromophore. It boasts a water content of 1.5%, aimed at minimizing glistening and surface irregularities [2, 10–12]. Leveraging this advanced material, the CNWT model was developed, incorporating TFNT’s optical design but constructed from CNA0T0. This iteration aims to enhance visual stability and contrast sensitivity while reducing subjective photic side effects compared to its predecessors [3]. It effectively minimizes glistening within the lens and surface-level silicone oil nanoglistening (SSNG) post-operation, preserving its initial transparency even nine years later [2]. Despite these advancements, studies comparing the Clareon IOL with the Acrysof IOL have shown similar rates of posterior capsule opacification [13], suggesting that material clarity alone may not account for the observed improvements in contrast sensitivity.

The AcrySof IQ and Clareon IOLs exhibit differences in their material composition and the design of their square edges [13]. The CNWT’s edge is more precisely defined compared to the TFNT, leading to a reduction in “edge glare.” This phenomenon, often experienced post-cataract surgery, manifests as halos or large semi-circles caused by light reflection [14]. The manufacturer has introduced a novel edge design for this material, implementing a refined posterior square optic edge aimed at decreasing positive dysphotopsias and inhibiting posterior capsule opacification (PCO) [15]. Furthermore, aspects of the IOL surface, such as the presence of glistenings and fold marks, have been identified as factors impacting contrast sensitivity [16, 17]. These issues may arise from the degradation of the lens surface, resulting from processes like hydrolysis, oxidation, and both enzymatic and physical wear [8]. Notably, SEM analysis revealed that the CNWT lens shape was more defined compared to the TFNT, correlating with improved contrast sensitivity in the CNWT. These findings align with research indicating the influence of IOL surface characteristics on contrast sensitivity.

The limitation of our study is that it is a short-term study, not a long-term evaluation of the effect of contrast sensitivity reduction on the IOL surface shape and lens edge. The previous report showed that CNWT has better contrast sensitivity than TFNT, but all are short-term evaluations [3]. On the other hand, it is difficult to evaluate long-term contrast sensitivity. There is a possibility that the gradual development of glistening occurs over a long period of time provides for the neuroplasticity mechanism to occur and compensate for the deteriorating visual acuity and quality of vision [18]. Future research including more detailed analysis of the mechanism is required.

## Conclusions

The SEM found that the lens shape of the CNWT was clear than that of the TFNT, and the contrast sensitivity of the CNWT was also higher than that of the TFNT. IOL surface features may affect contrast sensitivity.

## Data Availability

The datasets used and analysed during the current study are available from the corresponding author on reasonable request.

## Abbreviations

BCVA: Best-corrected visual acuity
cd: Candelas
CNA0T0: Clareon IOL, Alcon, Fort Worth, Texas, USA
CNWT: Clareon Panoptix, Alcon, Fort Worth, Texas, USA
cpd: Cycles per degree
CS: Contrast sensitivity
D: Diopters
IOL: Intraocular lens
SD: Standard deviation
SEM: Scanning electron microscope
SSNG: Sub-surface nano glistening
TFNT: Acrysof Panoptix, Alcon, Fort Worth, Texas, USA
